# M2 macrophages promote IL-10^+^B-cell production and alleviate asthma in mice

**DOI:** 10.1093/immadv/ltaf007

**Published:** 2025-03-10

**Authors:** Baichao Yu, Xueqi Wang, Yongkun zheng, Wenjun Wang, Xiaoqin Cheng, Yue Cao, Mingxing Wei, Ying Fu, Yiwei Chu, Luman Wang

**Affiliations:** Department of Immunology, School of Basic Medical Sciences, Fudan University, Shanghai, China; Department of Immunology, School of Basic Medical Sciences, Fudan University, Shanghai, China; Department of Immunology, School of Basic Medical Sciences, Fudan University, Shanghai, China; Department of Immunology, School of Basic Medical Sciences, Fudan University, Shanghai, China; Department of Neurology, Zhongshan Hospital, Fudan University, Shanghai, China; Department of Immunology, School of Basic Medical Sciences, Fudan University, Shanghai, China; Department of Gastroenterology and Hepatology, Zhongshan Hospital, Fudan University, Shanghai, China; Department of Immunology, School of Basic Medical Sciences, Fudan University, Shanghai, China; Department of Immunology, School of Basic Medical Sciences, Fudan University, Shanghai, China; Department of Immunology, School of Basic Medical Sciences, Fudan University, Shanghai, China; Department of Immunology, School of Basic Medical Sciences, Fudan University, Shanghai, China; Shanghai Fifth People’s Hospital, Fudan University, Shanghai, China

**Keywords:** asthma, IL-10+B cells, macrophage

## Abstract

**Introduction:**

B cells have a central regulatory role in various diseases. While macrophages are found in the disease microenvironment and interact with tissue and diverse immune cells, their relationship with B cells remains poorly explored.

**Methods:**

This study used an asthma animal model and macrophage depletion and demonstrated a significant exacerbation of asthma symptoms upon macrophage removal, coupled with a marked reduction in IL-10^+^ B-cell expression.

**Results:**

Further analysis revealed that the macrophages interacting with IL-10^+^ B cells in the asthma microenvironment were of the M2 subtype. Furthermore, our sequencing data indicated a potential mechanism wherein M2 macrophages promote IL-10^+^ B-cell activity through the TGF-β pathway and oxidative phosphorylation pathways.

**Conclusion:**

These findings suggest that M2 macrophages modulate IL-10^+^ B cells, ultimately mitigating asthma symptoms in mouse models.

## Graphical Abstract

**Graphical Abstract:**
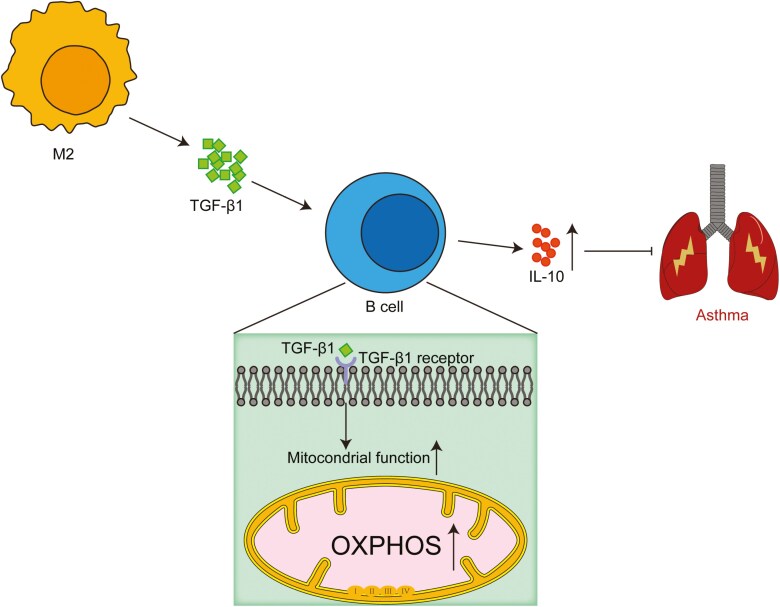

## Introduction

Regulatory B cells (Bregs) encompass all subsets of B cells that are crucial to suppressing immune responses [1, 2]. They are central to maintaining immune balance in physiological and disease states. While interleukin-10 (IL-10) is a hallmark for Bregs [3], their suppressive activities can also be mediated through the secretion of IL-10, IL-35 [4], and transforming growth factor β (TGF-β) [5] and the expression of cell surface proteins, including TIM-1 [6] and CD11b [7]. Recent studies have revealed that different inflammatory environments can induce distinct populations of Bregs [1, 8]. Although research has highlighted the significant regulatory function of Bregs in asthma [9–11], with a focus on understanding the mechanisms underlying their actions, how the inflamed pulmonary microenvironment influences their expansion remains to be explored. Therefore, there is a critical need for a deeper exploration of the microenvironmental factors that shape distinct Breg responses within the asthmatic lung.

Macrophages, as key players in the innate immune system, are ubiquitous in various tissues, contributing significantly to immunity, tissue repair, and overall homeostasis [12]. These versatile cells exhibit remarkable functional adaptability and can transition between different activation states in response to various environmental signals. The spectrum of macrophage polarization ranges from the “classically activated” M1 phenotype to the “alternatively activated” M2 phenotype, showcasing the diverse roles they can undertake [13, 14]. While diseases often involve the migration of multiple immune cell types to affected tissues where they interact with macrophages, the impact of macrophages on B cells remains poorly known.

Our study conducted on an asthma mouse model revealed that the lung microenvironment fosters the generation of IL-10^+^ B cells by M2 macrophages, improving the disease. This discovery sheds light on the intricate interplay between macrophages and B cells in the context of disease pathogenesis, highlighting a novel mechanism through which macrophages can influence B-cell responses for therapeutic benefits.

## Materials and methods

### Mice

C57/B6J(B6) mice were purchased from Shanghai Jihui Laboratory Animal Co., Ltd. (Shanghai, China). All mice were bred and housed in the animal facility of Fudan University (Shanghai, China) under specific pathogen-free conditions. The use of all mice in this study was approved by the Animal Care and Use Committee of the University of Fudan University.

### OVA-induced asthma model and macrophage depletion

For the OVA-induced asthma model, mice were intraperitoneally injected with 100 μl of OVA (50 μg, Sigma-Aldrich, USA)/Alum (Invitrogen, USA) on days 0 and 7. From day 14 to day 16, mice were anesthetized with isoflurane (RWD, China) followed by intranasal instillation of 50 μg OVA. On day 17, mice were anesthetized with 2% pentobarbital sodium, and serum, bronchoalveolar lavage fluid (BALF), and lung samples were collected for further analysis. For the depletion of lung microphages, 2 days prior to the induction of asthma, 60 μl Liposome Clodronate (FormuMax, USA) or PBS was administered intranasally every 4 days throughout the experiment.

*ELISA and Luminex*After centrifugation, the supernatant of BALF was collected for the detection of IL-4, IL-5, IL-13, and IL-10 by ELISA kits (Invitrogen, USA) following the manufacturer’s instructions. The collected serum was used for the detection of OVA-specific IgE and IgG1 as previously described [15].

Cell culture supernatant was harvested for the detection of BAFF, IL-1β, TNF-α, IL-6, IL-3, IL-10, IL-12, IL-18 by Luminex Assays kit (R&D, USA) and TGF-β1, IL-4, IL-13 by ELISA kits (Invitrogen, USA) following the manufacturer’s instructions.

### Lung histopathology

One lobe of the lung was fixed, embedded, and then sectioned at 5 μm thickness. The section was stained with Haemotoxylin and Eosin(H&E) or schiff periodic acid shiff. Subsequently, the stained sections were evaluated microscopically using the Leica DFC7000T microscope.

### Immunofluorescence

Paraffin sections of mouse lung tissue were stained with anti-mouse F4/80 (Abcam, USA), anti-mouse CD19 (Abcam, USA), and DAPI, following the instructions provided in the 4-color Fluorescence kit manual (Panovue, China). The stained sections were evaluated microscopically using the Leica DFC7000T microscope.

### Flow cytometry

Single-cell suspensions of BALF cells, digested lung cells as well as macrophages and B cells cultured in vitro were initially stained with anti-CD16/CD32 for blocking Fc receptor. Subsequently, cells were stained with surface markers and intracellular markers sequentially. For intracellular staining, cells were stimulated with Cell Stimulation Cocktail plus protein transport inhibitors (Invitrogen, USA) for 5 h before staining. Flow cytometry analysis was performed using BD FACSCelestraTM, and further data analysis was carried out using Flowjo V10 (BD, USA). Additionally, MitoTracker® Green FM or MitoTracker® Deep Red FM was used for the detection of mitochondrial mass and mitochondrial potential, respectively.

### Induction of bone marrow-derived macrophages and co-culture with B cells

Bone marrow cells were extracted by flushing the femurs and tibias of B6 mice. These cells were cultured in a complete DMEM medium (DMEM medium containing 10% fetal bovine serum, 1% penicillin, and streptomycin mixture) with 20 ng/ml M-CSF for 7 days. After 7 days, the medium was changed and either LPS 100 ng/ml + IFN-γ 10 ng/ml (M1) or IL-4 20 ng/ml+IL-13 20 ng/ml (M2) was added for further polarization. Subsequently, polarized macrophages and B cells that sorted by B cell isolation kit (Stemcell, Canada) were cocultured for 48 h. In certain experiments, the TGFβ1 blocking antibody or oligmycin or FCCP was introduced into the co-culture environment.

### Real-time reverse transcription PCR analysis

Total RNA was extracted using the RNAsimple Total RNA Kit (TIANGEN, China). and reverse-transcribed into cDNA with Hifair®II 1st Strand cDNA Synthesis SuperMix for qPCR (gDNA digester plus) (YEASEN, China) (YEASEN, China) according to the manufacturer’s instructions. The real-time PCR was performed on ABI7500 Thermocycler (Applied Biosystems, USA) in 20 µl reaction system containing cDNA, primers, distilled water, and Hieff UNICON® qPCR SYBR Green Master Mix (YEASEN, China). The reaction procedure was set according to the manufacturer’s recommended protocol. The 2-ΔΔCt method was employed to determine relative gene expression compared to β-Actin. The primer sequence for target genes is listed as follows:

**Table:** 

Gene	Primers (Sangon Biotech, Shanghai)
***Actin-F***	**CTACCTCATGAAGATCCTGACC**
***Actin-R***	**CACAGCTTCTCTTTGATGTCAC**
***Il6-F***	**CTTCTTGGGACTGATGCTGGTGAC**
***Il6-R***	**AGGTCTGTTGGGAGTGGTATCCTC**
***Il1b-F***	**TCGCAGCAGCACATCAACAAGAG**
***Il1b-R***	**AGGTCCACGGGAAAGACACAGG**
***Arg1-F***	**GGCAACCTGTGTCCTTTCTCCTG**
***Arg1-R***	**GGTCTACGTCTCGCAAGCCAATG**
***Ym1-F***	**GCCCACCAGGAAAGTACACAGATG**
***Ym1-R***	**GACCTCAGTGGCTCCTTCATTCAG**
***Atp6v1b2-F***	**CTATCCCAGCCTCGTCTCACCTAC**
***Atp6v1b2-R***	**TGCCCACTTCTCTTTGTGCCATC**
***Atp6v0c-F***	**GCAAAGTGACATGGCTGACATCAAG**
***Atp6v0c-R***	**ATGACTGACATGGCTGCGATGC**
***Atp6v1a-F***	**TCTATGTCGGCTGCGGTGAGAG**
***Atp6v1a-R***	**GGCATGTTGGAGGTGTTGGCTAC**
***Lhpp-F***	**AGCCACCTGCCAGATCCTGAAG**
***Lhpp-R***	**AGCATCCGCAATCACCACACAG**
***Atp6v0d2-F***	***AGCCAGCCTCCTAACTCAGCAG***
***Atp6v0d2-R***	***GAAGTTGCCATAGTCCGTGGTCTG***

### RNA sequence

The B cells sorted from the spleen were cultured with or without M2 macrophages for 24 h. Total RNA was extracted by RNAsimple Total RNA Kit (TIANGEN, China). Subsequently, RNA sequencing was carried out by the Beijing Genomics Institute (BGI, Shenzhen, China). The analysis was conducted on the platform of Dr. Tom (https://biosys.bgi.com/). The RNA-seq data have been deposited at GEO (https://www.ncbi.nlm.nih.gov/geo) with the accession number GSE249363.

### Oxygen consumption rates (OCR)

The OCR (Oxygen Consumption Rate) detection was conducted on unstimulated B cells, anti-CD40 stimulated B cells, and M2-educated B cells using the XF-96 Extracellular Flux Analyzer from Agilent, following the manufacturer’s guidelines.

### Statistical analysis

All statistical analysis was carried out using GraphPad Prism 8 (GraphPad Software). Statistical significance was assessed using the unpaired *t* tests, one-way ANOVA, and Pearson’s correlation. All results from cytometry assays are presented as the mean ± standard error of the mean (SEM).

## Results

### Interaction between lung B cells and macrophages in the asthma model

**Figure 1.**
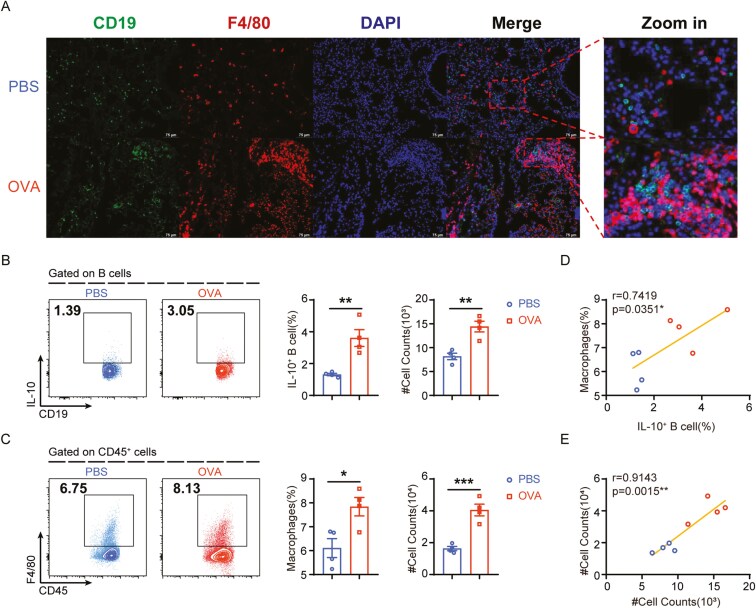
The potential crosstalk between IL-10^+^ B cells and macrophages in lung under asthmatic conditions. (A) IF staining of B cells and macrophages in lung from naïve mice following OVA or PBS treatment. Representative images are shown; scale bars, 75 μm. (B) Representative plots(left) and statistical results(right) of IL-10^+^ B cells in the lung of PBS and OVA group were shown. (C) Representative plots(left) and statistical results(right) of macrophages in the lung of PBS and OVA group were shown. (D) Correlation between macrophages and IL-10^+^B cell frequency in lung. (E) Correlation between macrophage and IL-10^+^B cell counts in lung. Data were shown as mean ±SEM and analyzed by unpaired *t* test (B, C) and Pearson’s correlation (D, E). Blots are representative of two or three independent experiments. *P < .05, **P < .01, ***P < .001, ****P < .0001. ns, no significant

Macrophages are the predominant immune cells within the lung and are central in the development of allergen-induced asthma [16]. Our immunofluorescence assays revealed a marked accumulation of macrophages in the lungs of asthmatic mice (Supplementary Fig. 1 and Fig. 1A). Particularly, we observed macrophages surrounding CD19^+^ B cells (Fig. 1A), hinting at a close spatial relationship between these two cell types. Building upon our previous findings that underscored the significant regulatory function of B cells in mucosal immunity [4, 7, 17], we conducted an in-depth analysis of lung B-cell and macrophage populations using flow cytometry. We observed an increase in the percentage of IL-10-expressing regulatory B cells (Bregs, CD19^+^IL-10^+^ B cells) compared with the control lungs (Fig. 1B). Concurrently, the macrophage frequencies also increased based on the conventional markers (Fig. 1C). Notably, a positive correlation was observed between the levels of regulatory B cells and macrophages in the lung (Fig. 1D–1E), indicating a potential local interplay between Bregs and macrophages within the pulmonary milieu under asthmatic conditions.

### Depletion of macrophages reduces Bregs

**Figure 2.**
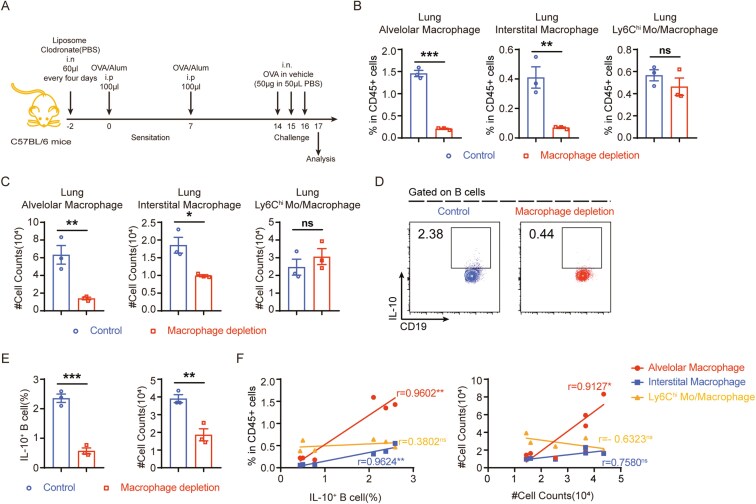
Macrophage depletion leads to a reduction in IL-10^+^ Bregs. (A) Schematic diagram illustrating the OVA-induced asthma model and macrophage depletion. (B) The proportion of different types of macrophages inlung of macrophage-depleted group and the control group. (C) The cell counts of different types of macrophages in lung of the macrophage-depleted group and the control group. (D-E) The proportion and cell count of IL-10^+^B cells in lungs of the macrophage-depleted group and the control group. (F) The correlation between the frequency and number of macrophages and B cells. Data were shown as mean ±SEM and analyzed by unpaired *t* test (B, C, E) and Pearson’s correlation (F). Blots are representative of two or three independent experiments. *P < .05, **P < .01, ***P < .001, ****P < .0001. ns, no significant

We initiated our investigation by depleting macrophages using liposome-encapsulated clodronate (liposome clodronate) before inducing asthma with ovalbumin (OVA) to explore the interplay between Bregs and macrophages and its potential impact on asthma pathophysiology (Fig. 2A). First, we confirmed that depleting macrophages effectively removed the alveolar and interstitial macrophages without impacting monocytes (Fig. 2B–2C, Supplementary Fig. 2A–2B). After macrophage depletion, asthma symptoms in mice worsened, as indicated by elevated total cell counts and increased levels of IL-5 and OVA-specific IgE in the bronchoalveolar lavage fluid (BALF), with more severe lung inflammation (Supplementary Fig. 3A, 3C–3E). Notably, reduced IL-10 expression was observed in the asthmatic group after macrophage depletion (Supplementary Fig. 3C). Further analysis focused on CD19^+^IL-10^+^ B cells, revealing a significant decrease in the percentage of CD19^+^IL-10^+^ B cells in the lungs after macrophage depletion (Fig. 2D–2E). Moreover, the expression of the CD19^+^IL-10^+^ B cells revealed a positive correlation with the macrophage expression levels (Fig. 2F). These findings suggest significant connections between macrophages and CD19^+^IL-10^+^ B cells in the context of asthma.

### M2 macrophages induce increased IL-10 expression production by B cells

**Figure 3.**
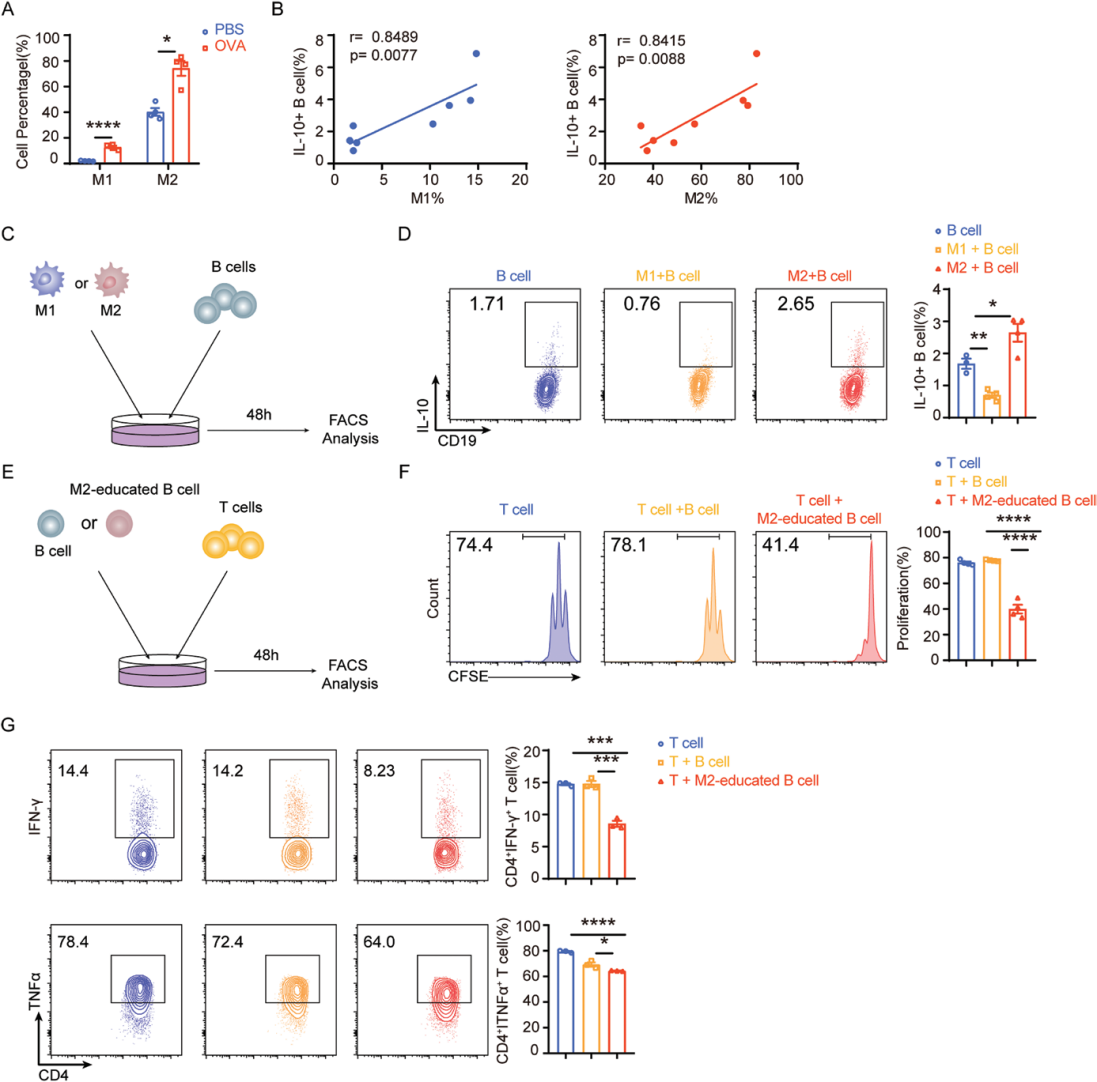
M2 macrophages induce enhanced IL-10 expression in B cells (A) The proportion of M1 and M2 macrophages in the lung. (B) The correlation between the frequency of M1/M2 macrophages and IL-10+B cell in the lung. (C) Schematic representation of co-culture between macrophages and B cells. (D) The proportion of IL-10^+^B cells in cultured B cells. (E) Schematic representation of co-culture between M2-educated B cells and T cells. (F) The proliferation (CFSE staining) of T cells with or without M2-educated B cell coculture was detected by flow cytometry. CFSE, 5,6-carboxyflu-orescein diacetate, succinimidyl ester staining. (G) The function (IFN-γ and TNFα) of T cells with or without M2-educated B cells coculture was detected by flow cytometry. Data was shown as mean ±SEM and analyzed by unpaired *t* test (A), Pearson’s correlation (B) and one-way ANOVA (D, F, G). Blots are representative of two or three independent experiments. *P < .05, **P < .01, ***P < .001, ****P < .0001. ns, no significant

Macrophages exhibit remarkable plasticity and can adopt different phenotypes based on environmental cues, playing diverse roles in health and disease. They can polarize into the proinflammatory “M1” or anti-inflammatory “M2” immunoregulatory phenotypes in response to various signals from microbes and tissues [13, 14]. In our asthma model, we observed an increase in CD19^+^IL-10^+^ B cells along with both types of macrophages during asthma attacks, with a positive correlation between the percentage of CD19^+^IL-10^+^ B cells and macrophages (Fig. 3A–3B). We induced the differentiation of M1 and M2 macrophages using specific stimuli—LPS + IFN-γ for M1 polarization and IL-4 + IL-13 for M2 polarization—to determine which type of macrophage promotes IL-10 secretion in B cells (Supplementary Fig. 4A–4C). Subsequently, we separately cocultured these polarized macrophages with naive B cells (Fig. 3C, Supplementary Fig. 5A–5D). Our findings revealed that M2-polarized macrophages could stimulate high IL-10 expression in B cells (Fig. 3D). Importantly, these M2-educated B cells demonstrated negative regulatory capabilities; they could inhibit T cell proliferation and effector functions. In contrast, M1-polarized macrophages did not induce B-cell differentiation into regulatory cells (Fig. 3E–3G). This observation suggests a critical role for M2 macrophages in promoting Breg responses with immunosuppressive functions, shedding light on a potential mechanism for immune regulation in asthma and offering insights into therapeutic strategies targeting these interactions.

### M2 macrophages induce regulatory B-cell differentiation via TGFβ1

**Figure 4.**
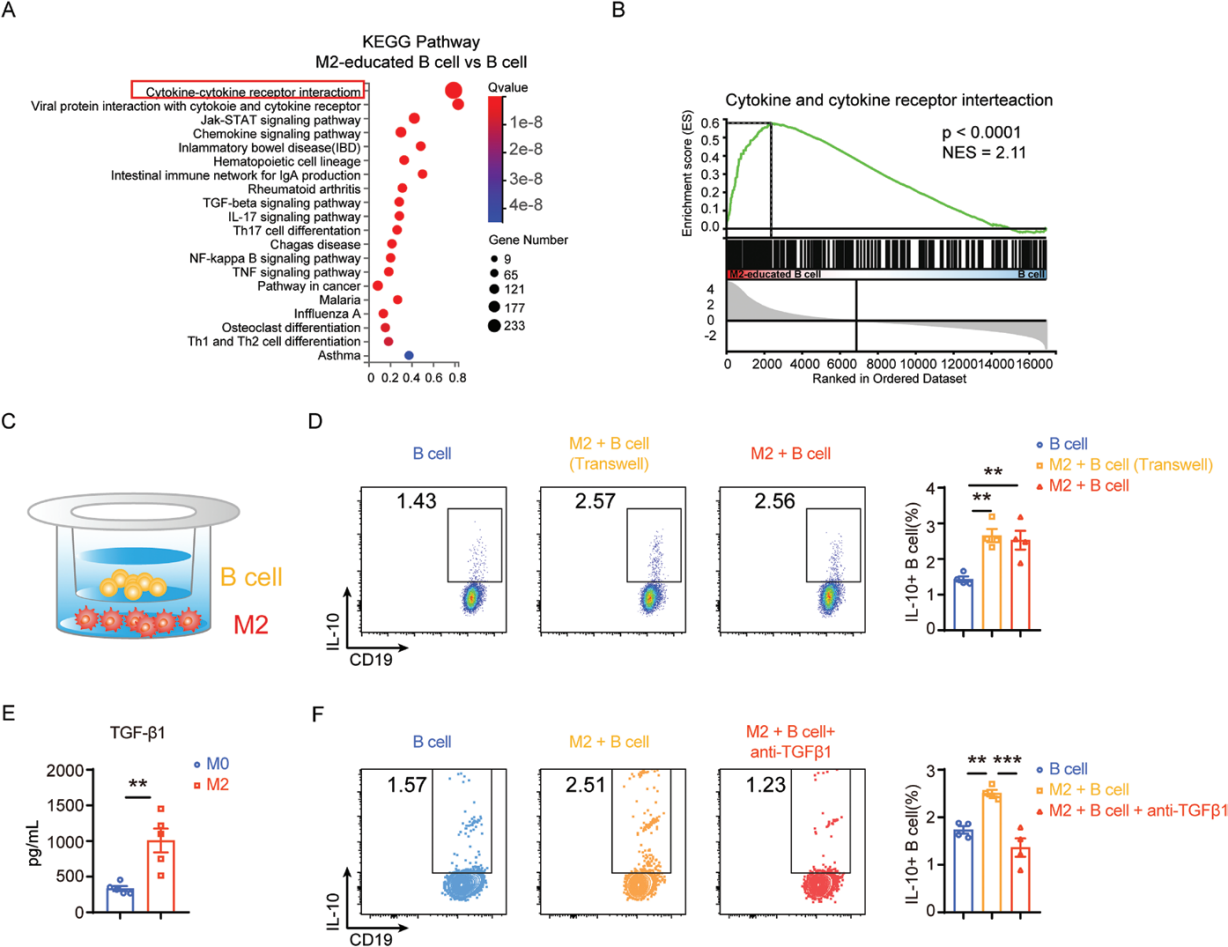
M2 macrophages promote IL-10^+^ Breg differentiation through TGFβ1. (A) KEGG gene enrichment analysis of up-regulated differentially expressed genes in M2-educated B cells compared to B cells (Cutoff: p.adj < 0.05; log2Foldchange >2). (B) Gene Set Enrichment Analysis (GSEA) of Cytokine and Cytokine receptor interaction pathway in M2-educated B cells compared to B cells. (C) Schematic representation of M2 macrophages and B cells co-culture using a transwell assay. (D) The proportion of IL-10^+^B cells in different conditions. (E) The detection of TGF-β1 expression in M0 and M2 macrophage culture supernatant using ELISA. (F) Flow cytometry analysis of IL-10+ B cells with or without anti-TGFβ1 monoclonal antibody in the presence of M2 macrophages. Data was shown as mean ±SEM and analyzed by unpaired *t* test (E) and one-way ANOVA (D, F). Blots are representative of two or three independent experiments. *P < .05, **P < .01, ***P < .001, ****P < .0001. ns, no significant

In our quest to delve deeper into the molecular mechanisms at play, we conducted RNA-seq analysis to compare the gene expression profiles of M2-educated B cells, naive B cells, and anti-CD40-stimulated B cells (Supplementary Fig. 5F–5I). The KEGG and GSEA analyses revealed significant enrichment in the cytokine-cytokine receptor interaction pathway (Fig. 4A and 4B). We used a transwell culture system that physically separated B cells and macrophages to confirm that this enrichment was mediated by cytokines (Fig. 4C). Intriguingly, the expression of IL-10^+^ B cells remained unaffected despite the absence of direct cell contact (Fig. 4D), suggesting a cytokine-mediated mechanism. Next, we utilized Luminex and ELISA techniques to screen M2-derived cytokines and determine which cytokine derived from M2 cells promoted IL-10 production in B cells. The results revealed a significant increase in TGF-β1 levels compared with other cytokines (Fig. 4E and Supplementary Fig. 5E). Subsequent blocking experiments further validated the pivotal role of M2-derived TGF-β1 in driving IL-10+ B-cell polarization (Fig. 4F). Our functional studies provide compelling evidence that M2 macrophages induce IL-10^+^ B-cell polarization through TGF-β1.

### M2 cells induce B-cell differentiation through the oxidative phosphorylation pathway

**Figure 5.**
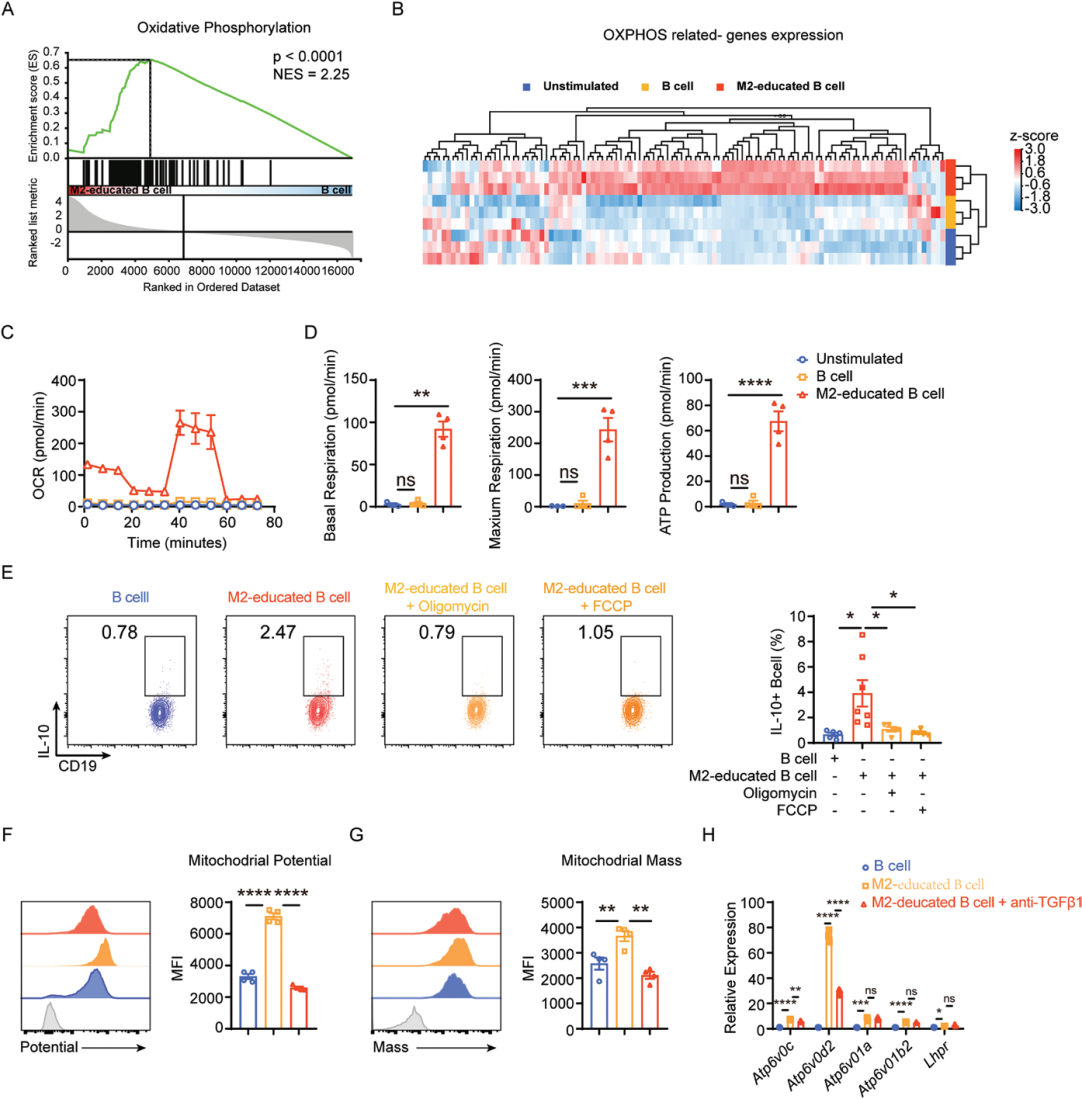
M2 macrophage-derived TGFβ1 may educate B cell differentiation into IL-10+ B cells through activating oxidative phosphorylation (OXPHOS). (A) Gene Set Enrichment Analysis (GSEA) of OXPHOS in M2-educated B cells compared to B cells. (B) Heatmap depicting OXPHOS-related gene expression between M2-educated B cells and B cells. (C) OCR of unstimulated-, anti-CD40 stimulated- and M2-educated B cells was detected using Seahorse analysis. (D) Statistical results of basal OCR, maximal OCR, and ATP production of unstimulated-, anti-CD40 stimulated- and M2-educated B cells were shown. (E) M2-educated B cells were co-treated with oligomycin or FCCP for 48 h, followed by the detection of IL-10^+^ B cells using flow cytometry. (F) Mitochondrial membrane potential was measured using MitoTracker® Deep Red FM. (G) Mitochondrial mass was measured using MitoTracker® Green FM. (H) qPCR for top five OXPHOS-related genes identified from RNA-seq. Data was shown as mean ±SEM and analyzed by one-way ANOVA (D-H). Blots are representative of two or more independent experiments. *P < .05, **P < .01, ***P < .001, ****P < .0001. ns, no significant

The GSEA analysis highlighted the activation of the oxidative phosphorylation (OXPHOS) pathway in M2-educated B cells (Fig. 5A) and revealed a significant upregulation in the expression of genes related to OXPHOS (Fig. 5B). Subsequent oxygen consumption rate measurements validated these findings, demonstrating that M2-educated B cells exhibited notably increased basal respiration, maximum respiration, and ATP production levels (Fig. 5C–5D). Additionally, inhibiting OXPHOS through oligomycin and FCCP significantly decreased IL-10^+^ B-cell production (Fig. 5E). These results suggest that M2 macrophages likely instruct B cells to differentiate into regulatory cells by activating the OXPHOS pathways.

**Figure 6.**
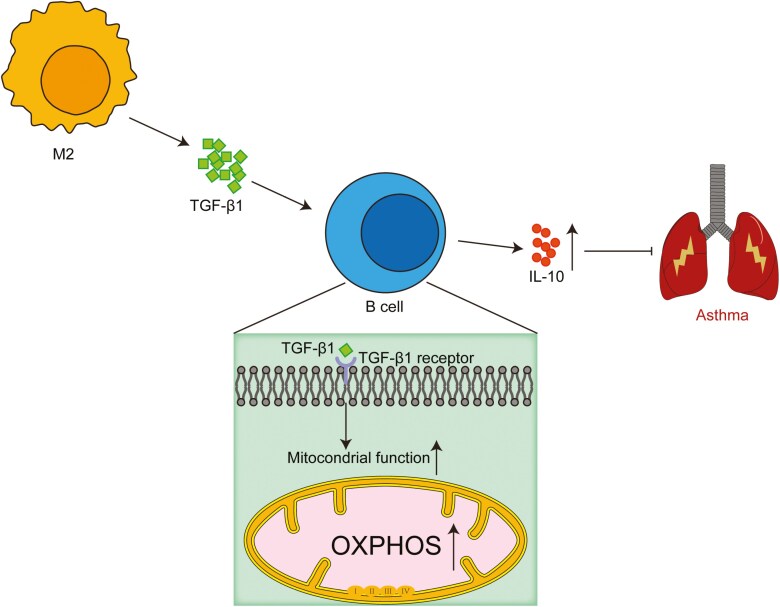
Schematic diagram summarizing the highlights of the study.

We assessed the mitochondrial potential and mass in M2-educated B cells with or without TGF-β1 monoclonal antibody using fluorescent probes to explore whether M2-derived TGF-1 facilitates the promotion of IL-10^+^ B cells via the OXPHOS pathway. Additionally, we examined the expression of the top five genes enriched in the OXPHOS pathway from our RNA-seq data via qPCR. The results indicated that blocking TGF-β1 mitigated the increase in mitochondrial potential and mass, along with the expression of OXPHOS-related genes (*Atp6v0c* and *Atp6v0d2*) in M2-educated B cells (Fig. 5F–5H). These findings indicate that M2 macrophages promote IL-10 production by enhancing OXPHOS in B cells through TGF-β1 production (Fig. 6).

## Discussion

Numerous studies have reported the interactions between macrophages and other cells in the tissue microenvironment. For example, in *Mycobacterium tuberculosis* infection, macrophages interact with CD8^+^ T cells in the BALF [18]. Besides, macrophages are critical in regulating the trafficking of neutrophils during inflammation by locally signaling to endothelial cells [19]. However, these reports on interactions between macrophages and B cells have primarily focused on how B cells educate macrophages and assist in their differentiation [7, 20, 21]; research on how macrophages educate B cells is limited. To the best of our knowledge, our findings fill a gap in the understanding of the interaction between macrophages and B cells in the disease environment, a crucial mechanism in alleviating asthma.

Bregs have immune suppressive functions mediated mainly by IL-10 production. Previous studies from our group and others have revealed that depleting Bregs results in more severe inflammation in colitis, pneumonia, and other inflammatory diseases affecting mucosal tissues [22, 23]. This observation highlights the crucial negative regulatory role of Bregs in maintaining immune homeostasis. In the context of allergic asthma, one of our first observations was the increased frequencies of Bregs in the lungs compared with the control mice, implying that Bregs could be involved in the lung immune response during asthma pathogenesis. Given their known anti-inflammatory properties, Bregs may work to dampen excessive Th2 inflammation to prevent immunopathology. Further investigation is warranted to define the precise differentiation and functions of Bregs in experimental asthma models.

Based on our previous results and literature reports, B-cell differentiation is highly influenced by the microenvironment. Factors like metabolism, microbiota, and other immune cells regulate B-cell differentiation toward regulatory phenotypes [7, 24, 25]. However, the role of macrophages as resident cells in inducing regulatory B cells remains unclear. In this study, we observed that macrophage levels varied with changes in Breg frequencies and that macrophage and B cells colocalized, suggesting that tissue-resident macrophages, as environmental sensor cells, may be inductive in regulating Breg. This finding offers a novel perspective worth further investigation to understand macrophage function in governing local immune responses through cellular crosstalk, including with Breg. More studies are needed to elucidate the underlying mechanisms and pathways through which macrophages may modulate Breg development or activity in health and disease contexts.

We aimed to deplete macrophages to demonstrate that they act on B-cell differentiation and subsequently influence disease progression. Several methods were reported for macrophage depletion, including clodronate liposomes [26], CSF1R inhibitors [27], and antibody-mediated depletion [28]. Among them, clodronate liposomes are the most commonly used. This method involves the intravenous administration of liposomes encapsulating clodronate, which induces macrophage apoptosis after phagocytose. However, some reports indicated that this method may cause neutropenia [29]. Therefore, we assessed the total cell counts and macrophage frequencies in the lung after clodronate liposome treatment. We found that alveolar and interstitial macrophages were significantly reduced, whereas monocyte levels remained unchanged. This observation demonstrates that macrophage depletion using clodronate liposomes was effective and specific in removing resident lung macrophages without affecting other immune cells. This method is reliable for studying the role of macrophages in our disease model.

M2 macrophages, also called alternatively activated macrophages, generally promote wound healing and tissue remodeling and have anti-inflammatory properties [30]. They are activated by signals like IL-4, IL-13, IL-10, and glucocorticoids rather than IFN-γ and LPS, which induce M1 activation. Common markers of mouse M2 macrophages include CD206, Arg1, Fizz1, and Ym1. In humans, CD163 and CD209 are used. Functionally, M2 macrophages secrete anti-inflammatory cytokines and growth factors that enhance angiogenesis and tissue remodeling and repair [31]. Emerging evidence suggests that M2 macrophages also influence adaptive immunity by inducing regulatory T and B cells in some contexts [32]. Targeting M2 macrophage polarization and functions is a potential therapeutic strategy for diseases driven by inflammation, infection, or fibrosis. Our research revealed that M2 macrophages promote IL-10^+^ B cells in asthma. With our current understanding of macrophages, targeting M2 cells for therapeutic intervention requires further investigation.

IL-10 is the hallmark cytokine produced by Bregs. However, the signaling pathway that induces IL-10^+^B cells may not necessarily be the IL-10-JAK-STAT pathway (predominantly the STAT3 pathway) [33]. Our previous studies also found that in the context of infection, reactive oxygen species promote the generation of regulatory B cells through one-carbon metabolism, contributing to establishing an unfavorable immune microenvironment during late-stage infection [23]. TGF-β, often derived from Tregs or APCs, induces Breg differentiation through SMAD and non-SMAD pathways [34]. Our study identified that the TGF-β secreted by M2 cells is the primary cytokine promoting the differentiation of IL-10^+^B cells through RNA-seq and transwell systems. TGF-β may promote IL-10+B-cell differentiation through the oxidative phosphorylation pathway.

Our discovery opens up new avenues for asthma treatment and offers potential therapeutic approaches by targeting the interplay between immune cells.

## Supplementary Material

ltaf007_suppl_Supplementary_Figures_S1-S5

## Data Availability

All data included in this study are available upon request by contact with the corresponding author.
