# Supplementary material for: M2 macrophages promote IL-10+B-cell production and alleviate asthma in mice
Source: Immunother Adv. 2025 Mar 10;5(1):ltaf007. doi: 10.1093/immadv/ltaf007 (PMC12059559; doi:10.1093/immadv/ltaf007)
Supplement: ltaf007_suppl_Supplementary_Figures_S1-S5 [file ltaf007_suppl_supplementary_figures_s1-s5.pdf]

Supplemental Figures

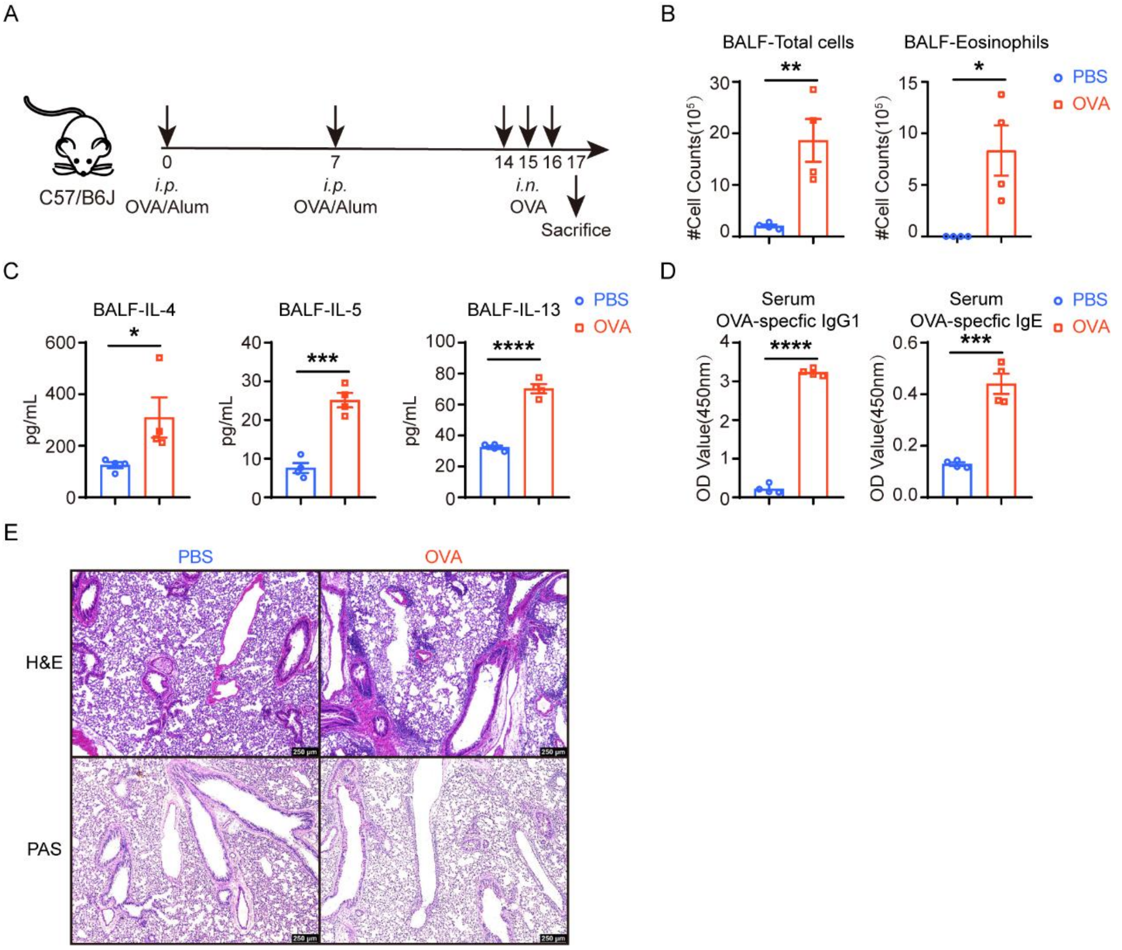

Supplementary Figure 1. The induction of OVA-induced asthma. (A) Schematic diagram. (B) The cell counts of total cells and eosinophils in the bronchoalveolar lavage fluid (BALF). (C) The detection of type II cytokines (IL-4, IL-5, IL-13) in BALF using ELISA. (D) The detection of OVA-specific IgG1 and IgE in serum using ELISA. (E) Representative histology images of lung section stained with H&E and PAS. Scale bar = 250μm. Data was shown as mean ±SEM and compared by unpaired t test. Blots are representative of two or more independent experiments. * $p < 0.05$, ** $p < 0.01$, *** $p < 0.001$, **** $p < 0.0001$. ns, no significant

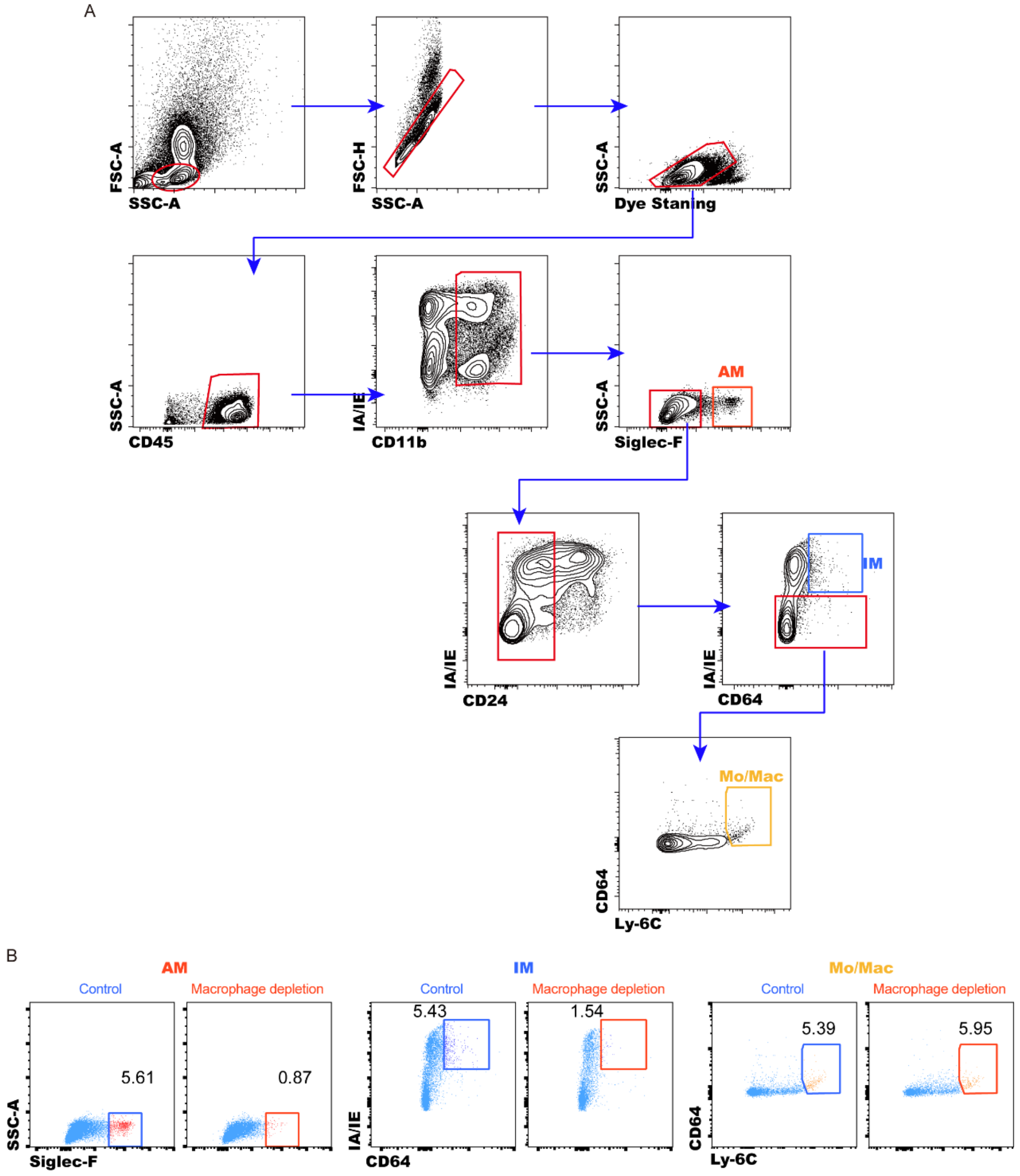

Supplementary Figure 2. The flow cytometry analysis of different macrophage subsets in lung following Liposome Clodronate treatment. (A) The gating strategy of lung macrophage classification. (B) Representative plots of different macrophage subsets in lung.

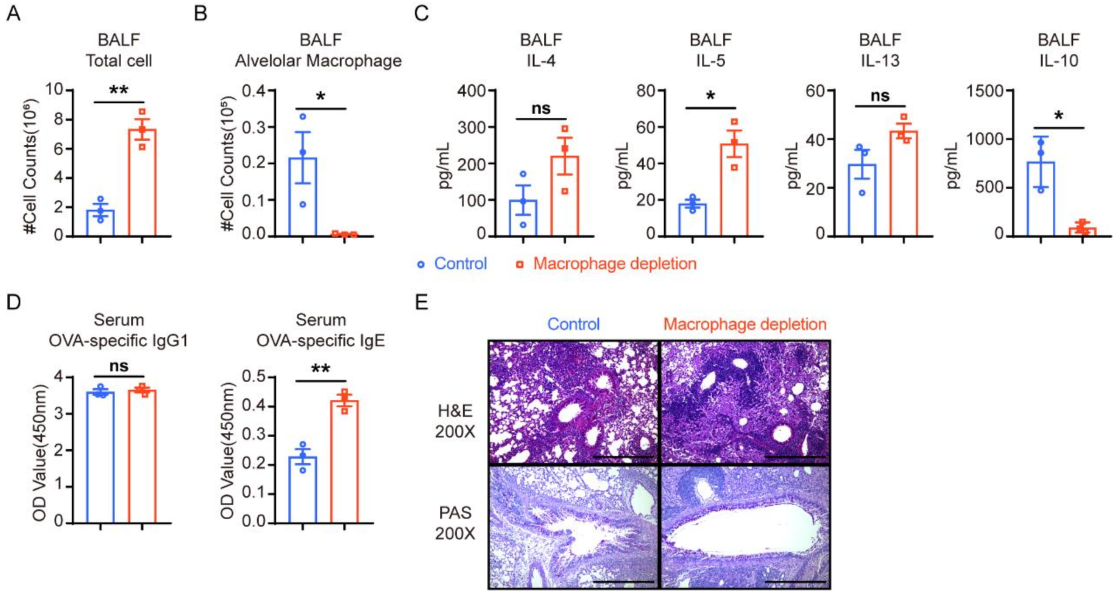

Supplementary Figure 3. The depletion of macrophages exacerbated OVA-induced asthma. (A-B) Total cell and alvelolar macrophage counts in BALF were measured by cell counter in macrophage-depleted and control group. (C) The concentrations of type II cytokines (IL-4, IL-5 and IL-13) and IL-10 in BALF were assessed using ELISA. (D) OVA-specific IgG1 and IgE levels in serum were assessed using ELISA. (E) Representative histology images of lung section stained with H&E and PAS. Scale bar=100μm. Data was shown as mean ±SEM and compared by unpaired t test. Blots are representative of two or more independent experiments. * $p < 0.05$, ** $p < 0.01$, *** $p < 0.001$, **** $p < 0.0001$. ns, no significant

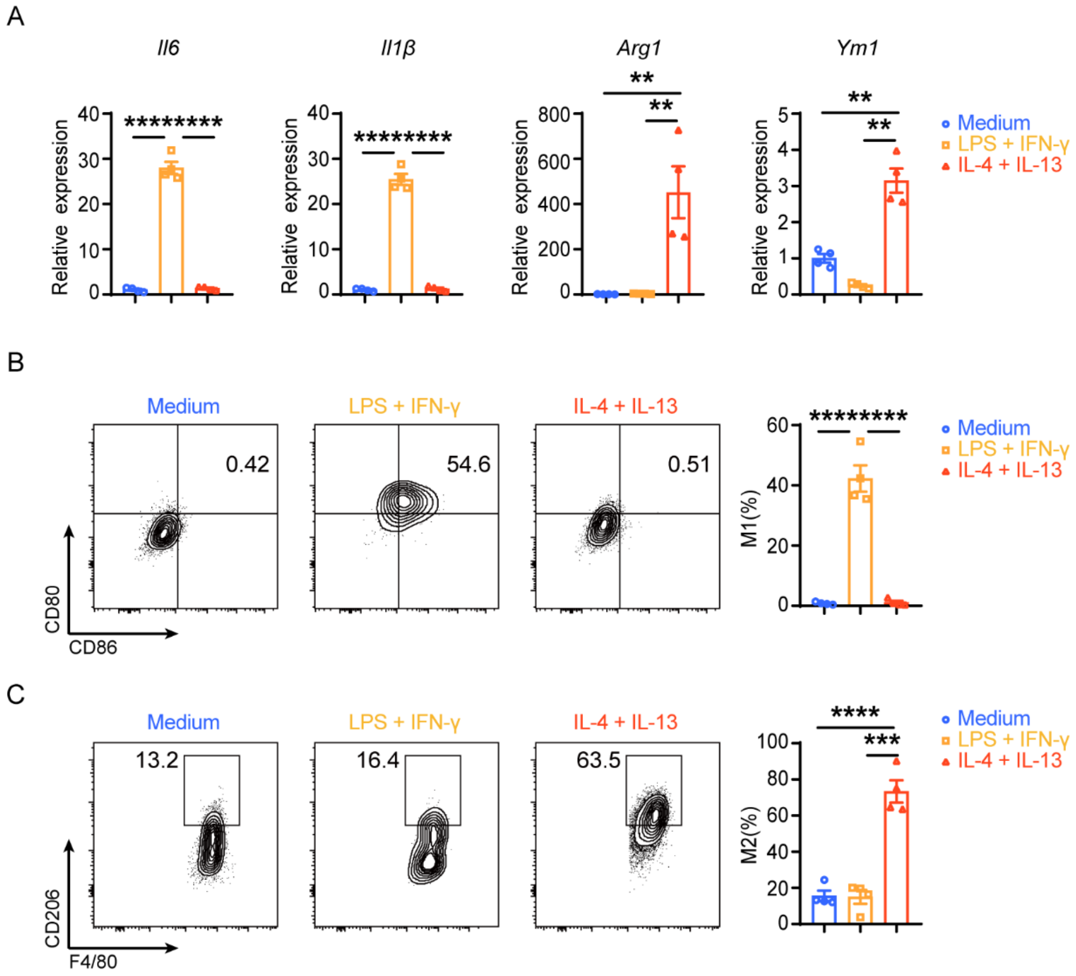

Supplementary Figure 4. The induction of M1 and M2 macrophages. (A) The mRNA expression of *Il6*, *Il1b*, *Arg1* and *Ym1* were measured by qPCR. (B) The frequency of $CD80^+CD86^+$ macrophages that represent M1 cell phenotype was determined by flow cytometry. (C) The frequency of $CD206^+F4/80^+$ macrophages that represent M2 cell phenotype was determined by flow cytometry. LPS + IFNγ represents stimulation for M1 polarization; IL-4 + IL-13 represents stimulation for M2 polarization; Medium represents M0 status. Data was shown as mean ±SEM and analyzed by one-way ANOVA. Blots are representative of two or more independent experiments. * $p < 0.05$, ** $p < 0.01$, *** $p < 0.001$, **** $p < 0.0001$. ns, no significant

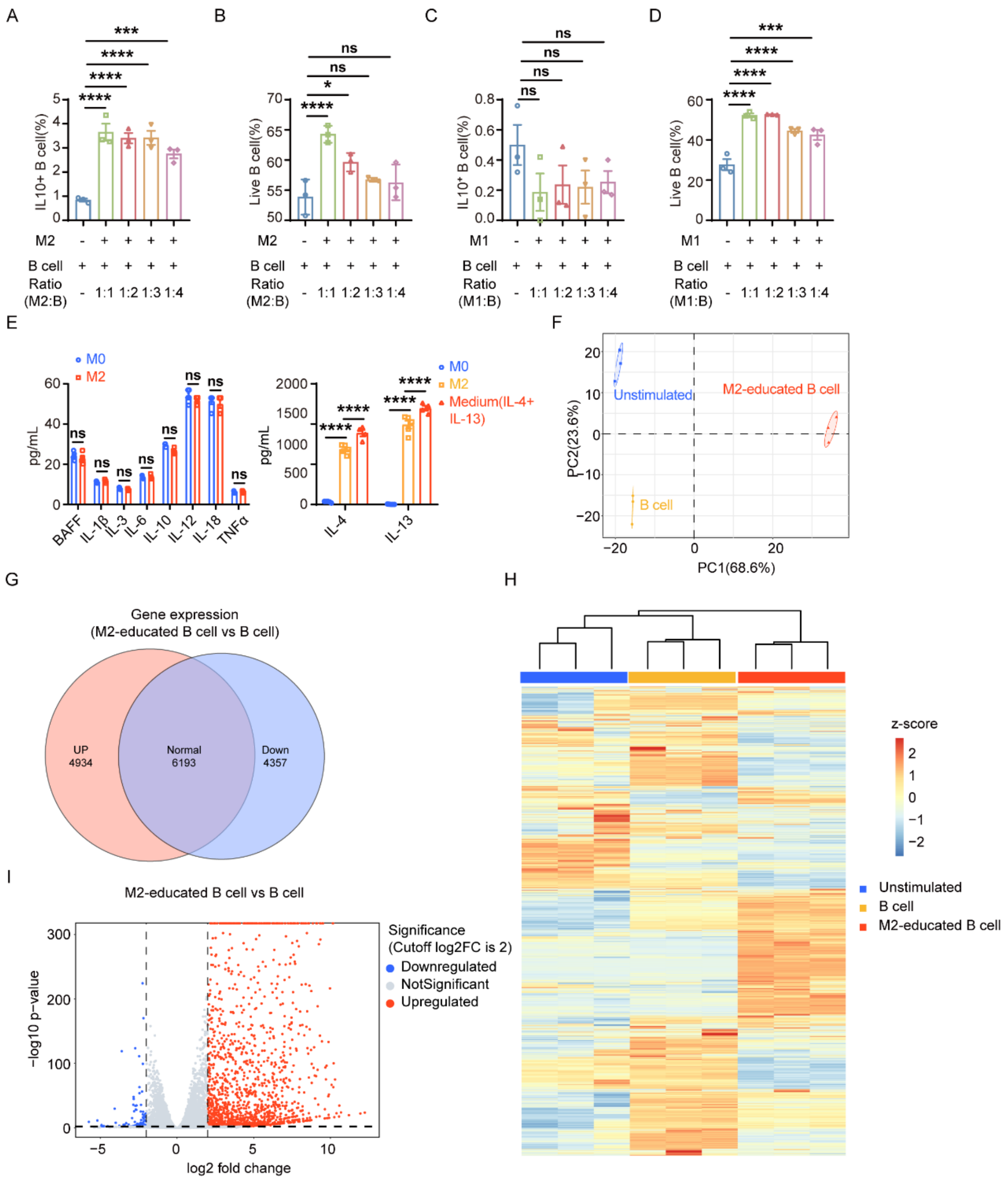

Supplementary Figure 5. RNA-seq revealed distinct gene expression patterns in M2-educated B cells compared to other experimental groups. (A) The percentage of IL-10⁺ B cells was assessed under varying ratios of M2 and B cell coculture conditions. (B) The viability of B cells was assessed under varying ratios of M2 and B cell coculture conditions. (C) The percentage of IL-10⁺ B cells was assessed under varying ratios of M1 and B cell coculture conditions. (D) The viability of B cells was assessed under varying ratios of M1 and B cell coculture conditions. (E) The different cytokines screen in supernatant of M0 and M2 culture groups using Luminex and ELISA. (F) Principal-component analysis plot of unstimulated-, anti-CD40 stimulated- and M2-educated B cells from RNA-seq data. (G) Venn diagram showing differential genes in M2-educated B cells compared to B cells

(Cutoff: p.adj < 0.05; log2Foldchange >0). (H) Heatmap depicting differential gene expression. (I) The volcano plot shows the differentially expressed genes in M2-educated B cells compared to B cells (Cutoff: p.adj < 0.05; log2Foldchange >2). Data was shown as mean ±SEM and analyzed by one-way ANOVA(a, b, c, d, e) and two-way ANOVA. Blots are representative of two or more independent experiments. * p < 0.05, **p < 0.01, ***p < 0.001, ****p< 0.0001. ns, no significant
